# Detection of T-2 Toxin in Wheat and Maize with a Portable Mass Spectrometer

**DOI:** 10.3390/toxins15030222

**Published:** 2023-03-16

**Authors:** Chris M. Maragos

**Affiliations:** Mycotoxin Prevention and Applied Microbiology Research Unit, National Center for Agricultural Utilization Research, Agricultural Research Service, United States Department of Agriculture, 1815 N University, Peoria, IL 61604, USA; chris.maragos@usda.gov

**Keywords:** mycotoxin, trichothecene, T-2 toxin, analysis, portable mass spectrometer, wheat, maize

## Abstract

T-2 toxin is a mycotoxin routinely found as a contaminant of cereal grains worldwide. A portable mass spectrometer was adapted to enable the detection of T-2 toxin in wheat and maize by APCI-MS. In order to facilitate rapid testing, a rapid cleanup was used. The method was able to detect T-2 toxin in soft white wheat, hard red wheat, and yellow dent maize and could be used to screen for T-2 at levels above 0.2 mg/kg. The HT-2 toxin was only detectable at very high levels (>0.9 mg/kg). Based on these results, the sensitivity was not sufficient to allow the application of the screening method to these commodities at levels recommended by the European Commission. With a cut-off level of 0.107 mg/kg, the method correctly classified nine of ten reference samples of wheat and maize. The results suggest that portable MS detection of T-2 toxin is feasible. However, additional research will be needed to develop an application sensitive enough to meet regulatory requirements.

## 1. Introduction

T-2 toxin belongs to a group of mycotoxins known as the trichothecenes. The trichothecenes are characterized by containing a six-member oxygen-containing ring, an epoxide, and an olefinic bond [1]. These toxins are produced by a group of fungal pathogens, the *Fusarium sambucinum* species complex, that commonly infest crops [2]. Plant diseases caused by fungi include Fusarium Head Blight and Fusarium Ear Rot [2]. Affected crops include wheat, barley, maize, oats, and other cereal grains. Fungal growth can result in the formation of T-2 toxin, which can cause disease in humans and animals. The trichothecenes are cytotoxic. They inhibit protein synthesis and mitochondrial function, can activate genes related to inflammation and can disrupt the function of the gastrointestinal system [3,4,5,6]. They are believed to have caused outbreaks of food poisoning between the 1940s and 1990s and have been associated with alimentary toxic aleukia (ATA) [6,7,8]. Symptoms of ATA include emesis, diarrhea, leukopenia, anemia, bone marrow depletion, and bleeding from the nose and mouth [6,7]. It has also been suggested that there may be a link between chronic exposure to trichothecenes and Kashin-Beck disease, a form of degenerative osteoarthritis [6].

**Figure 1:**
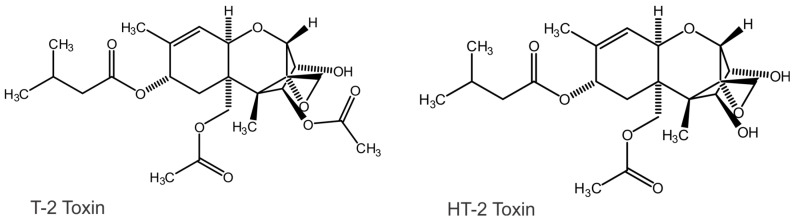
Structures of T-2 toxin and HT-2 toxin.

Animals and plants can metabolize the T-2 toxin, often removing the C-4 acetate and thereby yielding the HT-2 toxin (Figure 1). Both compounds can undergo further metabolism, including de-acylation, de-epoxidation, or conjugation through phase II metabolism. Glycosylation is one such outcome, yielding T-2 glucosides as modified forms [9,10,11]. T-2 toxin has an absorption maximum in the deep ultraviolet (UV) (191 nm) and infrared absorptions at 3400, 2940, 1635, and 1240 cm^−1^ [12,13]. It has not been reported to be fluorescent. The trichothecenes are relatively heat stable, with a treatment of 120 °C for 55 min causing 7% breakdown. A treatment of 217 °C for 35 min caused 95% degradation [14,15,16,17].

Detection and measurement of T-2 have been accomplished using technologies ranging from immunoassays and biosensors to LC-MS. Rapid screening assays for T-2 toxin have existed for many years, and several are available commercially in formats such as microplate enzyme-linked immunosorbent assays (ELISAs) and immunochromatographic devices, also known as ‘dipsticks’, ‘test strips’, and lateral flow immunoassays [18]. Recently such strips have been adapted to test for multiple mycotoxins that include T-2 toxin [19,20,21]. Antibody-based biosensors for T-2 and HT-2 have also been reported [22,23,24], as well as a fluorescence polarization immunoassay [25] and aptamer-based tests [26,27]. Current trends in immunoassays for mycotoxin detection were reviewed by Nolan et al. [28].

The immunoassays can be used to screen for the presence of toxins, and if confirmation is needed, samples can be re-tested using chromatographic techniques. Having an absorbance of 191 nm has meant that many commercial solvents, which contain stabilizers that absorb in this range, can not be used. However, UV detection is enabled with appropriate solvent selection, such as with high-purity acetonitrile [12]. T-2 toxin can also be measured by HPLC following attachment with a fluorescent label [29,30,31]. Numerous mass spectrometric (MS) based techniques have also been applied, including GC-MS and various forms of LC-MS. In the case of GC-MS, the toxins were derivatized to improve volatility for measurement [32,33,34]. Early reviews on the analysis of T-2 toxin and its metabolites were provided by Meneely et al. [35] and Krska et al. [36]. The number of LC-MS methods that have been applied to T-2 and HT-2 detection is enormous, and the methods reported are quite diverse. Reviews on the use of LC-MS-based methods include those by Zhang et al. [37] and De Girolamo et al. [38]. Several multi-mycotoxin LC-MS/MS (LC-MS2) methods have undergone collaborative study [39,40,41,42,43] and are widely accepted as the techniques of choice for regulatory compliance. Most such methods use mixtures of acetonitrile and water for extraction. Recently an eco-friendly LC-MS2 method for T-2 and HT-2 was reported that used ethanol/water (4 + 1 *v*/*v*) for extraction, followed by immunoaffinity column (IAC) cleanup and reverse phase LC with aqueous ethanol [44].

The MS methods that have been reported for T-2 toxin have used laboratory-based instruments and are not truly portable. Recently, however, a portable MS instrument with an electrospray ionization (ESI) source was used to measure another group of mycotoxins, the fumonisins, in maize [45]. A rapid, portable, MS-based method has not been previously reported for T-2 toxin, which, unlike the fumonisins, is amenable to detection following atmospheric pressure chemical ionization (APCI). The objective of this research was to develop a rapid MS-based method for T-2 toxin in wheat and maize using a portable scanning mass spectrometer equipped with APCI. In order to accomplish this objective, the APCI source of a commercial instrument was modified to permit the introduction of sample extracts by infusion. The modified source was used to apply flow-injection analysis mass spectrometry to the detection of T-2 toxin in wheat and maize.

## 2. Results

### 2.1. Modification of the APCI Source

**Figure 2:**
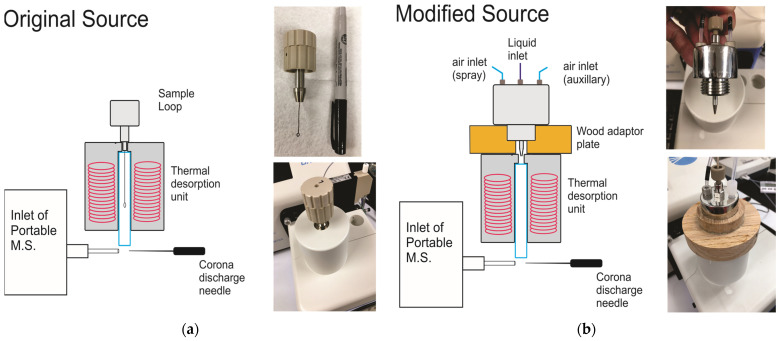
Diagrams and photos of the original (unmodified) and modified APCI sources. (**a**) original source, with sample loop pictured (**b**) source modified to include a spray inlet. An aquarium pump was used to supply air for the spray and auxiliary inlets. The rightmost photos show a closeup of the spray inlet and mounting of the spray inlet onto the thermal desorption unit.

A truly portable mass spectrometer is one in which the instrument can be easily transported, can be rapidly set up, and which has minimal external requirements so that it can be used in a variety of non-laboratory situations. The instrument chosen for this research weighs 10 kg (22 lb), does not require an external source of gas (nitrogen or helium), and requires only a single 110 V electrical connection. The electrical requirement is the only aspect that keeps it from being fully ‘field portable’. The latter could be remedied with either a large battery and inverter or by using a portable generator. The instrument was purchased with an APCI source, a schematic of which is shown in Figure 2a. The original source was designed so that samples would be introduced using a metal sample loop dipped into the test material (liquid or solid) and then placed into the thermal desorption (TD) unit for volatilization. In preliminary experiments (data not shown), this form of sample introduction was used to test wheat flour (as a solid), crude extracts of wheat flour, purified extracts, and toxin standards in various solvents. Our preliminary results suggested that signals could be obtained at relatively high toxin concentrations in a solvent but not in crude grain extracts or the grain itself. Furthermore, the nature of the sample introduction resulted in a transient signal that was difficult to reproduce. In the original form, the source did not appear to be well suited for the rapid detection of low levels of mycotoxins in commodities.

The source was modified to function more as a ‘traditional’ APCI source, that is, one that would allow a continuous flow of solution to be sprayed into the TD unit. This was achieved by fitting the spray nozzle from an older Thermo Finnigan LCQ mass spectrometer (Figure 2b). To maintain portability, a battery-powered aquarium pump was used to provide the spray gas (air). The design was amenable to connection with an HPLC pump. However, to keep the system portable, samples were infused with a simple syringe pump.

### 2.2. Responses of the Portable MS and Matrix Effects

**Figure 3:**
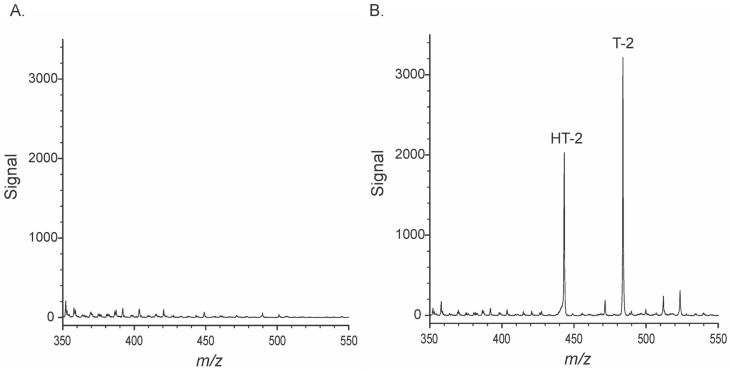
Partial spectra were collected in positive mode APCI using the modified source and the portable MS: (**A**) solvent, with no added toxin, and (**B**) with T-2 and HT-2 toxins at 0.5 and 2 µg/mL, respectively. Labels indicate the [M + NH_4_]^+^ adducts. Full spectra were collected over the range of *m*/*z* 50-850.

The modified source was initially tested with T-2, and HT-2 was added to solvent or added to extracts of wheat. Previous experience with ambient ionization of T-2 and HT-2 using Direct Analysis in Real Time (DART) had suggested that the ammonium adducts of the toxins would be the most likely forms seen [46]. This is consistent with reports from many other laboratories using ESI sources [19,39,41,44,47,48,49,50]. Preliminary results (not shown) suggested that ionization was inhibited in solvent mixtures containing acetonitrile but feasible with solvent mixtures of aqueous methanol (MeOH). To facilitate the formation of the ammonium adduct, NH_4_COOH was incorporated as well. Spectra of T-2 and HT-2 in 12.5 mM NH_4_COOH in H_2_O/MeOH (2 + 1 *v*/*v*) are depicted in Figure 3.

T-2 and HT-2 toxins are generally extracted from cereal grains with aqueous mixtures of acetonitrile or MeOH [39,40,41,43,47,49,50]. Cleanup of the extracts before chromatography can be accomplished in several ways, involving either solid phase dispersion techniques such as QuEChERs, solid phase extraction (SPE) columns, or immunoaffinity columns (IAC) [19,41,43,44,48,49,50]. With very sensitive instrumentation, ‘dilute-and-shoot’ approaches can also be used [40,47]. The IAC provides a very clean extract that was previously shown to work well for detecting fumonisins in maize [45]. However, the cleanup is lengthy and requires a higher level of skill than simple solid phase adsorption columns such as the MycoSep column. When the extract is passed through the latter, many of the impurities bind to the column while the toxins pass through, making it a very rapid (approximately one minute) cleanup. The downside to this approach was that MeOH was not compatible with the MycoSep columns, which have been optimized for use with an acetonitrile/H_2_O (84 + 16 *v*/*v*) mixture. This was unfortunate because, as noted previously, ionization of T-2 and HT-2 in the presence of acetonitrile was severely inhibited.

**Figure 4:**
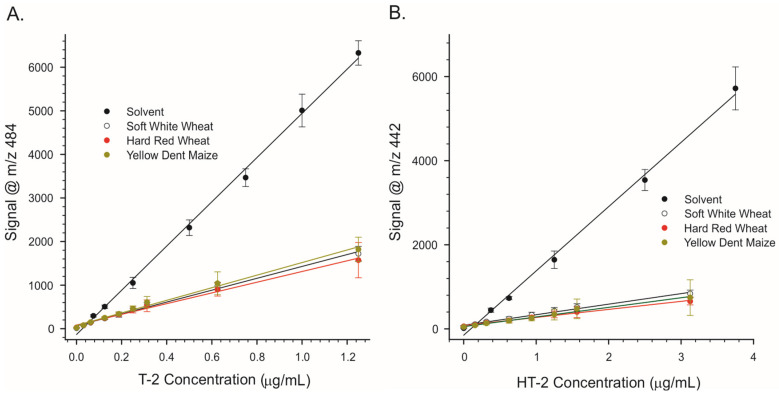
Raw (non-transformed) responses of the portable APCI-MS to trichothecene mycotoxins. The toxins were infused in the solvent, in purified wheat matrices, or in purified yellow dent maize matrix. (**A**) T-2 toxin. (**B**) HT-2 toxin.

In order to overcome this, the cleaned-up acetonitrile/water extracts were taken to dryness under nitrogen, which allowed the samples to be transferred into aqueous MeOH containing NH_4_COOH. This step added about 20 min to the procedure: 15 min for the dry-down and 5 min for reconstituting and filtering the extract. However, even with this modification, the method was still more rapid than using an IAC, which can require 30 min or more to perform [45]. In order to determine the extent to which the remaining matrix affected ionization, calibration curves were prepared in a purified matrix and in solvent (12.5 mM NH_4_COOH in 2 + 1 H_2_O/MeOH). The results are depicted in Figure 4.

**Figure 5:**
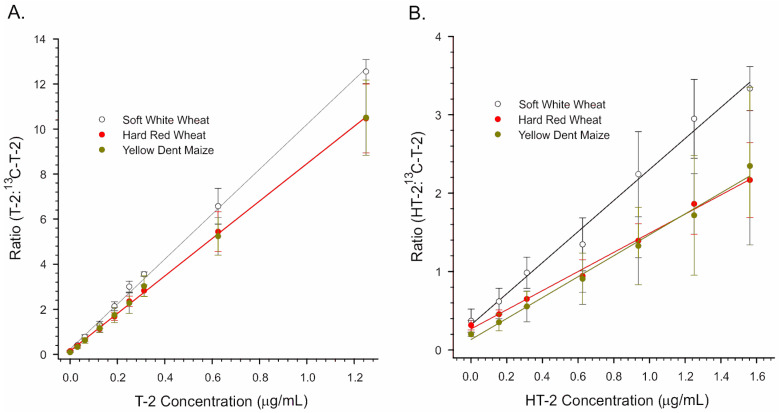
Calibration curves for (**A**) T-2 toxin and (**B**) HT-2 toxin added into the purified matrix from two types of wheat and yellow dent maize. Data presented as the ratio of signals found for the indicated toxin to that of ^13^C-T-2 added as an internal standard. Fits of the regression lines are presented in Table 1.

Clearly, even extracts that had been purified still caused severe inhibition of ionization. With ‘raw’ (non-transformed) signal data, the effects of extracts of soft white wheat, hard red wheat, and yellow dent maize were similar. To control matrix effects, the stable isotope ^13^C-T-2 toxin was incorporated as an internal standard into the solvent mixture used to reconstitute the dried extracts. Figure 5 depicts results presented as a ratio of the signal of either T-2 or HT-2 to that of ^13^C-T-2. Fits of the linear regression lines depicted in Figure 5 are provided in Table 1. For both T-2 and HT-2, the slopes of the regression lines were greater in extracts of soft white wheat relative to hard red wheat or maize. This suggests slightly better responses in soft white wheat compared to the other two matrices.

The data from Figure 5 and Table 1 were used to calculate the limits of detection (LOD) and quantification (LOQ) in each matrix. The LOD was calculated from the control extracts plus three standard deviations from the average of the controls. The LOQ was calculated from the control extracts plus 10 standard deviations. Values for the LOD and LOQ in each of the three matrices are presented in Table 2. One aspect that is readily apparent is the much better response of the TD-APCI to T-2 toxin relative to HT-2 toxin. LODs were almost two orders of magnitude greater for HT-2 than for T-2.

### 2.3. Cut-Off Values in Hard Red Winter Wheat

**Figure 6:**
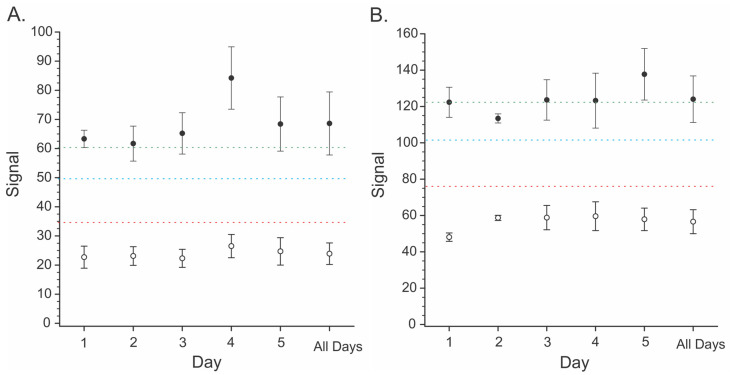
Responses of the portable MS to hard red winter wheat, either un-spiked (open circles) or spiked (closed circles) with 0.2 mg/kg T-2 and 1.5 mg/kg HT-2. Each point represents four samples ± one standard deviation (SD), except the points for “All Days”, which represent 20 samples. The red line indicates the calculated LOD (3 SD), the blue line is the cut-off value, and the green line is the LOQ (10 SD). (**A**) T-2, (**B**) HT-2.

The portable MS instrumentation was evaluated using criteria established by the European Commission for screening assays for T-2 and HT-2 toxins [51]. This involved testing eight samples (four spiked, four un-spiked) on each of five days for a total of 40 samples (20 spiked, 20 un-spiked). Spiking was performed at 0.2 mg/kg (T-2) and 1.5 mg/kg (HT-2). The average signal from the un-spiked samples and associated error estimates were used to calculate the LOD and LOQ for each of the toxins in hard red winter wheat. Data in Figure 6 depict the signals from each group of samples over five days and the average from all five days (“All Days”). In the case of the T-2 toxin, the spiking level was slightly above the calculated LOQ, while for the HT-2 toxin, the spiking level was very near the LOQ. The cut-off value is the signal at which there is a 5% chance of a false negative result. For T-2, this corresponded to a signal of 49.9, equivalent to 0.107 mg/kg in wheat. For HT-2, this corresponded to a signal of 101.9, equivalent to 1.215 mg/kg in wheat. Unlike the LOD and LOQ, the cut-off values are calculated from the average of the spiked samples and associated error estimates. The separation between the cut-off value and the spiking levels indicates that the spiking levels were well above those yielding a five percent false negative rate. This suggests that the screening test ‘passed’ at the spiking levels that were used.

### 2.4. Performance with Reference Materials

**Figure 7:**
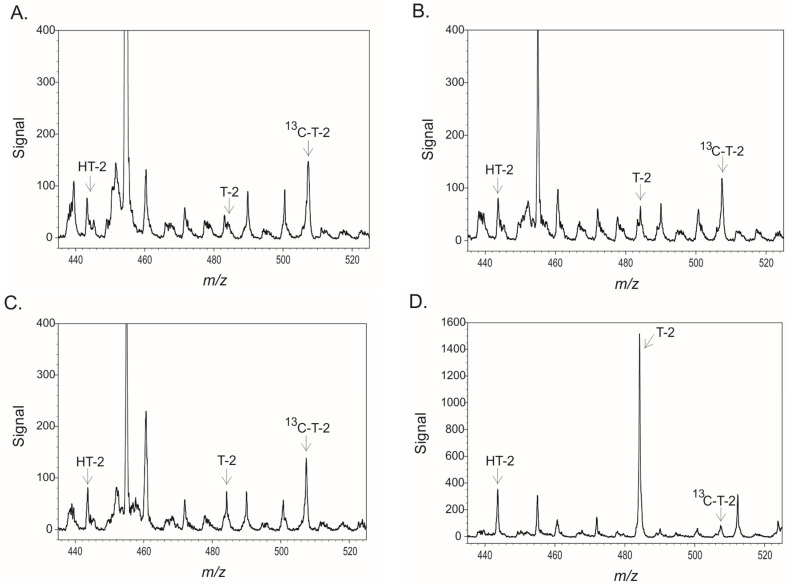
Spectra of four reference materials. (**A**) wheat sample TCQ-MMA12-100 batch 121236 with non-detectable T-2, (**B**) wheat sample TCQ-MMA12-100 batch 121235 with T-2 near the cut-off value, (**C**) maize sample TR-MT100 batch MTC-9999D with T-2 near the cut-off value, (**D**) highly contaminated maize sample (11,400 µg/kg T-2 and 500 µg/kg HT-2).

The portable MS method was tested with 10 samples of grain containing established amounts of T-2 and HT-2 toxins. Six of these (four samples of maize and one sample each of wheat and oat) contained T-2 levels greater than the cut-off value (0.107 mg/kg). The remaining four samples, all wheat, were samples reported by the supplier as having less than 0.005 mg/kg of T-2 or HT-2. The negative samples were all correctly classified as negative (Table 3, Figure 7). All the samples of maize and wheat that were above the cut-off for T-2 were correctly classified as ‘suspect’. A sample of oat flour containing T-2 near the cut-off was incorrectly classified as ‘negative’. All 10 of the reference samples had indicated HT-2 levels below the cut-off value (1.215 mg/kg) and, therefore, should have been classified as negative. However, two of these, containing more than 0.4 mg/kg, were classified as suspect (Table 3). Therefore, despite the high cut-off value for HT-2, the method did not permit accurate classification for this toxin.

## 3. Discussion

The original APCI source was designed to accept liquid samples in small volumes introduced into the thermal desorption (TD) unit with a sample loop. The modified source allowed liquid samples to be sprayed into the TD unit, with air as the source of the spray gas. T-2 and HT-2 toxins were readily detectable in positive mode APCI. The instrument responded much better to T-2 than to HT-2 (Figure 2). This was evident when the ratios of the slopes from the calibration curves for T-2 and HT-2 were compared. The ratios ranged from 4.9 (soft wheat) to 6.8 (hard red winter wheat). There was also a roughly five-to-seven-fold difference in the LODs for the two toxins in grain matrices (Table 2). The difference between the detection of T-2 and HT-2 in APCI is not a unique observation, and several reports had also shown better sensitivity for T-2 than for HT-2 when ESI was used [44,48,49,50]. We have also observed a similar effect previously with the ambient ionization technique of DART-MS [46].

The poorer response of the portable MS to HT-2 was significant, as the European Commission regulates both toxins in grains [52]. The cause for the poorer signals from HT-2 is a matter of speculation. It may be a result of lower volatility and/or ionization. HT-2 lacks the C-4 acetate present in T-2 (Figure 1) and may be less volatile. However, this did not prevent Lattanzio et al. [53] from developing an LC-APCI-MS/MS method for measuring the combination of T-2 and HT-2 after enzymatic hydrolysis. That method attained limits of detection of 0.4 to 0.5 µg/kg, which were well below those achieved here with the portable MS. In part, this may have been due to the reduction in matrix effects that were provided by including a chromatographic step. It was also likely a result of using a more sensitive, laboratory-based mass spectrometer with MS/MS capability. With respect to the portable MS used here, samples were infused (no chromatography), and there was a matrix component with an *m*/*z* similar to that of [HT-2 + NH_4_]^+^. As a result, the background signal for HT-2 toxin and the associated standard deviation in this signal in the matrix was much greater than for T-2 toxin. This itself was enough to negatively impact the LOD and LOQ for HT-2, which, of course, are based on these statistics. Attempts to tune the instrument to better detect HT-2 came at the expense of the detection of T-2 (data not shown). The tune parameters selected were done to optimize the detection of both. Nevertheless, the detection of HT-2 was poor.

Even after cleanup through a MycoSep column, the residual matrix from soft white wheat, hard red wheat, and yellow dent corn was enough to cause severe ion suppression (Figure 4). In part, matrix effects could be offset using ^13^C-T-2 as an internal standard (Figure 5). In the case of hard red winter wheat and yellow dent corn, the LODs of 0.02 to 0.028 mg/kg were sufficiently low to detect T-2 at relevant levels (Table 2). The EC criteria specify individual performance criteria for T-2 and HT-2 semiquantitative screening methods (Annex II of Commission Regulation No 519/2014). The goal of such methods is to allow the selection of those samples with levels of mycotoxins that exceed a Screening Target Concentration (STC). Those above the screening level are considered “suspect”, while those below are considered “negative”. A cut-off level can be calculated from the response of samples spiked at the STC. The cut-off value establishes the signal at which there is a five percent rate of false negatives, as it is critical that samples deemed “negative” truly contain less toxin than the STC. In situations where a regulatory level exists, the STC is equal to the regulatory level.

For natural toxins, the allowable levels are generally based upon a balance between the levels that are considered toxicologically significant and the levels that can be readily achieved with good agronomic practices. The European Food Safety Authority (EFSA) provides assessments used by policymakers in setting acceptable levels within the European Union (EU). An assessment of T-2 and HT-2 toxins established a tolerable daily intake of 0.02 µg/kg body weight per day and an acute reference dose of 0.3 µg [54]. In this context, the EU has set indicative levels (violative levels) ranging from 15 µg/kg in cereal-based foods for infants to 2 mg/kg in oat milling products [52]. In its current form, the portable MS method had LODs for T-2 that fell within the upper end of this range. For HT-2 they were, for practical purposes, outside this range. In the experiments with the portable MS, the STC was selected as 200 µg/kg for T-2 and 1500 µg/kg for HT-2. These values were chosen based on expectations for the sensitivity of the assay toward these toxins. Experimental results demonstrated that the cut-off levels were below these STC and hence acceptable for screening at these levels. However, the relatively high STCs used suggest that the method will likely only be applicable to certain commodities where the indicative levels are high (such as in compound feeds). While the portable MS was able to measure HT-2, in a practical sense, it did so only at very high levels. It is concluded that the portable MS method described herein is not suitable for detecting either toxin in cereal products for human consumption within the EU. The method may be suitable in situations where the screening level is set higher than the EU regulatory limit. In the United States, where no regulatory levels exist for T-2 and HT-2 toxins, there may be utility in screening to identify wheat or maize containing greater than 0.2 mg/kg T-2.

The practical application of the portable TD-APCI-MS at levels that meet the full range of EU regulatory requirements requires further improvements in sensitivity. Perhaps this will come with improvements to the linear ion trap upon which the instrument is based. Currently, that appears to be a significant technical challenge. As an alternative, the required sensitivity may be attained by using an instrument capable of MS/MS. Transportable MS/MS instruments are available but are significantly bigger and heavier (less portable) than the instrument used herein. Reducing the size of MS/MS instruments may ultimately solve the issues that were encountered here. An alternative solution may be to use a small, portable chromatography system to introduce samples. The resulting decrease in matrix interferences would likely contribute to improved sensitivity. Reduction of the matrix could also be achieved with a different form of sample preparation: for example, immunoaffinity cleanup, which was used successfully for the measurement of fumonisins in corn [45]. However, the downside of more extensive cleanup can be greater costs, longer sample processing times, and greater difficulty performing assays in non-traditional laboratory settings. For this reason, sample preparation will also be a key issue for improving portable MS-based methods for mycotoxins.

## 4. Conclusions

A portable mass spectrometer was adapted to enable the detection of T-2 and HT-2 toxins in wheat and maize by APCI-MS. In order to facilitate rapid analysis of these toxins, a rapid cleanup was used. The method was able to detect T-2 and HT-2 in soft white wheat, hard red wheat, and yellow dent maize. The method can be used to screen for T-2 at levels that are greater than 0.2 mg/kg. However, sensitivity was not sufficient to allow the application of the method to the screening of these commodities at levels recommended by the European Commission. Attaining the sensitivity to meet such criteria will likely require further advances in instrumentation, such as portable MS/MS, improvements in sensitivity to the linear ion trap used here, or the incorporation of a more extensive cleanup, such as an immunoaffinity column cleanup.

## 5. Materials and Methods

### 5.1. Materials

Except where noted otherwise, deionized water (Nanopure II, Thermo Scientific, Waltham, MA, USA) was used in the preparation of all reagents. HT-2 toxin in solid form was obtained from Sigma Chemical, dissolved to approximately 1 mg/mL in ethanol, and the concentration was established by comparison to an HT-2 analytical standard supplied by Biopure (Tulln, Austria). In a similar fashion, solid T-2 toxin was dissolved to approximately 2.5 mg/mL in acetonitrile, and the concentration was established by comparison to a T-2 analytical standard supplied by Trilogy Analytical Laboratory (Washington, MO, USA). Standard solutions of ^13^C-T-2 toxin were purchased from Biopure. Reference materials of wheat or maize containing established levels of T-2 and HT-2 toxins were also purchased from Trilogy. These included four samples of wheat flour with non-detectable T-2 and HT-2 (LOD 0.005 mg/kg), one sample of wheat flour contaminated with both toxins, and four samples of ground maize contaminated with both toxins. A single sample of contaminated oat flour was obtained from FAPAS (York, UK). Details of these samples are listed in Table 3. For spiking studies and for evaluation of matrix effects, samples of whole wheat berries were purchased from a local grocery (Sunrise Health Foods, Peoria, IL, USA) and online from Palouse Brand (Palouse, WA, USA). Samples included Hard Red Winter Wheat and Soft White Wheat and purchased in November 2022. All other chemicals were reagent grade or better and purchased from major suppliers.

### 5.2. Extraction and Cleanup

Samples of whole-kernel wheat or whole-kernel maize were ground to the consistency of flour using a coffee grinder. Twenty-five grams was extracted with 100 mL of acetonitrile/water (84 + 16, *v*/*v*) by shaking in a wrist-action shaker (Burrell Corporation, Pittsburg, PA, USA) for 30 min. The solution was filtered through a Whatman 2V filter (Cytiva, Buckinghamshire, UK). Then 4 mL of extract was applied to a Romer MycoSep 225 cleanup column, and approximately 2.5 mL of cleaned-up extract was collected. Of this, 1 mL of cleaned-up extract was dried under a gentle stream of nitrogen gas for 15 min at 50 °C. The dried extract(s) were reconstituted with 2 mL of a solution of 12.5 mM ammonium formate prepared in H_2_O/MeOH (2 + 1, *v*/*v*). In instances where ^13^C-T-2 was incorporated as an internal standard, the solution also contained 0.1875 µg/mL of the stable isotope. Reconstituted extracts were filtered through a 0.2 µm pore nylon filter, 13 mm diameter (Cytiva). The purified extracts contained the equivalent of 0.125 g of grain per mL of extract.

The extent of matrix on ionization was determined by preparing calibrants in ‘solvent’ (12.5 mM ammonium formate in H_2_O/MeOH, 2 + 1) or in purified extracts of uncontaminated wheat or maize. Calibrants were prepared over the range of 0.03125 to 1.25 µg/mL T-2 and 0.15625 to 6.25 µg/mL HT-2. Experiments on matrix effects were conducted in quadruplicate. Spiking was performed by adding standard T-2 and HT-2 in acetonitrile to ground wheat or maize. Spiked samples were stored overnight at ambient temperature to allow the solvent to evaporate prior to extraction. For validation of screening tests, the criteria of the European Commission [51] specify testing of a minimum of four samples per day, each, of spiked and un-spiked (control) material, with testing conducted on five separate days. To this end, 20 samples of control hard red winter wheat and 20 samples of the same wheat spiked with 0.2 mg/kg T-2 and 1.5 mg/kg HT-2 were prepared and analyzed.

### 5.3. Instrumentation

The mass spectrometer is a model ‘Portability’ instrument manufactured by BaySpec (San Jose, CA, USA). The instrument contains a linear ion trap mass analyzer and contains an inlet suitable for atmospheric sampling. It is self-contained in the sense that it does not require an external supply of gas, as air is used. The source that was purchased was a thermal desorption atmospheric pressure chemical ionization (TD-APCI) unit. The original source was equipped with a single route for sample introduction consisting of a metal inoculation loop (Figure 2a). The loop resembles those commonly used in microbiology to inoculate media and can hold a small volume of sample solution (circa 2 µL).

The inlet of the original APCI source was modified to accept a continuous supply of infused liquid. The spray nozzle from the API source of a Thermo Finnigan LCQ DECA mass spectrometer (San Jose, CA, USA) was used for this purpose (Figure 2b). The spray nozzle was affixed to the inlet of the thermal desorption unit via a wood adaptor plate. Spray gas and auxiliary gas consisted of air introduced with a portable aquarium pump: Hurricane Category 5 model 500 (Pet Tek International llc, Diamond Bar, CA, USA). The unit had two air outlets, each providing 2.05 L of air per min and was operated in Continual mode. One outlet was connected to the spray inlet and the other to the auxiliary inlet of the nozzle. The liquid inlet of the nozzle was connected via PFTE tubing to a syringe pump (New Era Pump Systems, Inc., Farmingdale, NY, USA). The spray capillary was 100 µm i.d., 350 µm o.d. from Millipore-Sigma (Burlington, MA, USA). The flow rate was 80 µL/min.

Operating parameters for the mass spectrometer were as follows: positive mode; heater: 200 °C; sample pump: 0% (note, this is the mass spectrometer’s internal sampling pump, not the air pump used for spraying); ISM HV: +2.8KV, ISM HVS Anode: −1200V. Settings for the mass analyzer were as follows: mass center: *m*/*z* = 484; mass tolerance: 1; spectrum average: 10, with running average enabled. Spectra were collected over the *m*/*z* range from 50 to 850. The instrument was tuned using purified T-2 and HT-2. The finalized tune parameters were: AC start: 3.0; AC stop: 28.0; AC frequency: 362.5; AC phase: 82; Rf level: 100; Trap bias: 2.5; Trap entry: 4; and Trap exit: 32. Spectra were collected in both Profile and Centralized modes. Signals from *m/z* 484 were used to determine T-2, and signals from *m/z* 442 were used to determine HT-2.

### 5.4. Data Analysis, Limits of Detection, Quantitation, and Cut-Off Value

In some experiments, such as for determining the extent of matrix effects, ^13^C-T-2 toxin was incorporated as an internal standard at a concentration of 0.1875 µg/mL. In such cases, relative response ratios were obtained by dividing the signals from T-2 or HT-2 by the ^13^C-T-2 signal. Calibration curves were constructed using either the un-ratioed (“raw”) data or the relative response ratios to ^13^C-T-2. Data were fit using a first-order linear regression (TableCurve, SYSTAT Software, Inc., Richmond, CA, USA). The limits of detection (LOD) were determined as the concentration of toxin calculated to give a response three standard deviations above that of control (un-spiked) samples. The limit of quantitation (LOQ) was determined as the concentration calculated to give a response 10 standard deviations above that of control samples.

For hard red winter wheat, an additional set of experiments were conducted to determine the Cut-Off value. The Cut-Off value represents the signal at which the rate of false negatives is predicted to be five percent. It was determined according to guidelines developed by the European Commission [51] and was based on Equation (1):

Cut-Off value = R_STC_ − t-value_0.05_ × SD_STC_ (1)

The screening target concentration (STC) was the concentration at which the assay would be used to screen samples. In this case, the STCs were 0.2 mg/kg (T-2) and 1.5 mg/kg (HT-2). Twenty samples spiked at this level were used to obtain an average response for each toxin (R_STC_). The one-tailed t-statistic (t-value_0.05_) for 20 replicates was 1.729. The SD_STC_ was the standard deviation in the signal ratios for the 20 spiked samples.

## Figures and Tables

**Table 1:** Fit of the linear regression lines depicted in Figure 3.

Toxin	Matrix	Linear Regression	r^2^
T-2 Toxin	Soft White Wheat	Y = 0.233 + 9.667 × [T-2]	0.9983
	Hard Red Wheat	Y = 0.155 + 8.307 × [T-2]	0.9995
	Yellow Dent Maize	Y = 0.152 + 8.300 × [T-2]	0.9988
HT-2 Toxin	Soft White Wheat	Y = 0.321 + 1.980 × [HT-2]	0.9896
	Hard Red Wheat	Y = 0.267 + 1.220 × [HT-2]	0.9950
	Yellow Dent Maize	Y = 0.134 + 1.334 × [HT-2]	0.9905

**Table 2:** Limits of detection (LOD) and quantification (LOQ) in terms of levels in commodities before extraction.

Toxin	Commodity	LOD in Matrix (mg/kg)	LOQ in Matrix (mg/kg)
T-2 Toxin	Soft White Wheat	0.028	0.27
	Hard Red Wheat	0.020	0.1
	Yellow Dent Maize	*	0.08
HT-2 Toxin	Soft White Wheat	2.03	6.30
	Hard Red Wheat	1.51	4.30
	Yellow Dent Maize	0.91	2.13

* NA: calculated value was negative. LOD was calculated as the concentration estimated to give a signal 3 standard deviations above the background. LOQ was calculated from 10 standard deviations above the background.

**Table 3:** Limits of detection (LOD) and quantification (LOQ) in terms of levels in commodities before extraction.

Material Type	Product Code	Reference [T-2 Toxin] (µg/kg)	T-2 Qualitative Result on the Portable MS (±)	Reference [HT-2 Toxin] (µg/kg)	HT-2 Qualitative Result on the Portable MS (±)
Maize	TR-MT100 Batch MTC-9999D ^1^	263.7 ± 59.6	Suspect	523.3 ± 83.9	Negative ^3^
Maize	TQC-MMA11-100 Batch 122298 (MM) ^1^	330.3 ± 23.3	Suspect	449.3 ± 33.7	Suspect
Maize	TR-MT100 Batch T-C-970 ^1^	324.0 ± 67.9	Suspect	414.5 ± 45.3	Negative
Maize	Not annotated ^1^	11,400	Suspect	500	Suspect
Wheat	TR-MT100 Batch MT-W-ND ^1^	ND @ 5	Negative	ND @ 5	Negative
Wheat	TR-MT100 T-W-975 ^1^	ND @ 5	Negative	ND @ 5	Negative
Wheat	TQC-MMA12-100 Batch 121236 (MM) ^1^	ND @ 5	Negative	ND @ 5	Negative
Wheat	TCQ-MMA12-100Batch 121252 (MMND) ^1^	ND @ 5	Negative	ND @ 5	Negative
Wheat	TQC-MMA12-100 Batch 121235 (MM) ^1^	230.4 ± 24.1	Suspect	183.5 ± 21.9	Negative
Oat Flour	T2296QC ^2^	149 ± 32	Negative	187 ± 39	Negative

^1^ Obtained from Trilogy Analytical Laboratory, ^2^ Obtained from FAPAS, ^3^ Negative: below the cut-off value (0.107 mg/kg for T-2, 1.215 mg/kg for HT-2); Suspect: above the cut-off.
